# Tell me a story

**DOI:** 10.7554/eLife.50527

**Published:** 2019-08-06

**Authors:** Joshua R Sanes

**Affiliations:** 1 Center for Brain ScienceHarvard UniversityCambridgeUnited States; 2 Department of Molecular and Cellular BiologyHarvard UniversityCambridgeUnited States

**Keywords:** point of view, writing, scientific publishing

## Abstract

Many authors start with the figures when writing a scientific paper, but it is easier to tell a good story if you start with the Introduction and the Results, and leave the figures to later.

Think back to when you were a little girl or boy, going on a long drive or getting ready for bed. From time to time, you probably asked a parent to tell you a story or read you a storybook. I know I did and so did my children. Well, we’re big boys and girls now, but we still love stories. Scientists can take advantage of this basic human desire by incorporating elements of storytelling when they prepare articles for submission to scientific journals: articles that tell a story will be better understood by and have a greater impact on readers.

Of course, scientific story telling is not easy. Aside from the fact that few scientists are trained in writing, there are two major problems. First, we have to tell the truth, a restriction story-tellers do not face. Second, we have to deal with restrictive formats, such as length limits, figure limits, and mandated order of sections. Nonetheless, it can be done and done well.

I think that the key is to prepare your paper in the sequence that a storyteller would. The three most important parts of a paper are the figures, the Results and the Introduction, probably in that order. It is therefore no surprise that authors often begin by preparing the figures, then move on to Results, and save the Introduction for near the end. In this article I will explain why I think it is better to reverse this sequence.

## Maybe the histogram could go under the micrograph

Starting with the text is not as strange as it might seem. Take operas for example: even though they are all about the music, the libretto is usually written before the score. That said, I do understand why it has become common practice to prepare figures before writing the text. It is easier and more fun to make figures than to think or write. After a day of cropping micrographs, adjusting font sizes, and arranging the panels in perfect rectangles, you feel like you’ve gotten something done. But all this is a displacement activity (definition: an unnecessary activity that you do because you are trying to delay doing a more difficult or unpleasant activity).

This is not to say that you should ignore your data at this stage: you absolutely need to know what you have and don’t have before you write. A simple expedient is to collect rough versions of panels with minimal editing on a digital bulletin board – PowerPoint slides work well. Then write the text and revise it a few times before arranging and polishing the figures. In my lab, and despite my pleas, people often bring me excellent figures along with fragments of text or no text at all. As we go through draft after draft, panels are often added, removed or altered – and always rearranged. It makes me feel bad to think about the wasted time and effort.

Don’t think I’m trying to overturn long-held dogma. The 'figure first' strategy is a product of Photoshop and Illustrator. Back in the day, when micrographs were generated with an enlarger and graphs with Letraset, it was necessary to have a definitive plan before starting to print and draw. I have no nostalgia for those cumbersome methods – but they did help make sure that thought preceded action.

## Our aim was to test…

So if not with the figures, where should you start? With the plot. You likely began your study with a question in mind. What does gene w do? How does cell x develop? Can method y help us understand disease z? At some point, you feel that you have gained enough insight to begin writing a paper – but more often than not, the data don’t provide an answer to the precise question you began with. If you try to fit the answer to the question, you risk ending up with a compendium of results that is less cohesive than it could be. Instead, start with the answer, figure out what the question should have been, and build on that. This seems counterintuitive, but it works. It is the first step in crafting a story.

The key is to prepare your paper in the sequence that a storyteller would

Once you have the question and answer in mind, write a rough draft of the Introduction, treating it as what is now called an elevator-pitch – a succinct statement meant to convince the listener/reader of your product’s value. What was the gap in knowledge you wanted to fill? What is the question to which you will provide an answer? Why is it important? What was your strategy? What did you find out? What conclusions did you draw? Why did they matter?

## Next we asked…

Using the introduction as a guide, move on to the results. Think hard about the best order in which to present them, feeling free to take advantage of what is, to my knowledge, the only fiction that is fully allowable in a scientific paper: you don’t need to present experiments in the sequence in which they were done or explain why they were actually done. Put another way, a scientific paper is not an autobiography; the story you tell should be about the science, not about you. The order of presentation can be, and often should be, quite different from the order of execution.

To organize the results, begin with a detailed outline in which you take account of the data you have. Your PowerPoint repository will be valuable here. As the outline takes shape, you will likely find some holes that you need to fill. If you’re lucky, you’ll also find some datasets that seemed worrisomely incomplete but don’t matter now, because they are not essential to the story you are going to tell. Revise the outline to take account of these realizations.

Next, write a draft of the Results section. Then read it over and reconsider whether you’ve made it easy for the reader to understand how the results lead to the conclusions you want to draw. If they don’t, you can rearrange sections, consider changing the plot, or even come to grips with the possibility that you’re not as close to finished as you had hoped to be.

## The whole truth

In presenting your results, you have to tell the truth and nothing but the truth. What you don’t have to do is tell the whole truth. In other words, you can select the results you present, as well as the order in which you present them, to shape your narrative. There is one crucial caveat: if you have results that call your conclusions into question, you need to present them, and explain why your (possibly modified) conclusions are still justified. My point is that you don’t need to describe everything you did. Think about whether each group of results makes the story more compelling or serves as a distraction. If it is the latter, be ruthless in omitting it. On occasion, you may have spent so much time on a set of experiments that you just can’t bear to cut it out completely. Try to resist temptation, but if you can’t, make it short.

Even when describing the most relevant results, work on being concise. This is difficult, as noted long ago by Blaise Pascal in an aphorism generally credited to Mark Twain: “I would have written a shorter letter but I didn’t have the time.” Just as it takes effort to omit distracting results, it takes effort to edit out needless detail. A few weeks ago, I tried to read an article in my field that seemed like a lightly edited lab notebook. I bet it was full of useful information, but I’ll never know. There was no story there so I quickly put it aside.

## Our main conclusions are…

Next comes the Discussion, which provides an opportunity for you to highlight what you want the reader to remember – the key results and principal conclusions. This is conventionally done by summarizing the results in a paragraph, following with sections devoted to major points, and finishing with a brief concluding paragraph. This format works well if you keep the story in mind as you write.

Think about whether each group of results makes the story more compelling or serves as a distraction. If it is the latter, be ruthless in omitting it.

To that end, remember that just as you don’t have to include marginally relevant data in the Results section, you don’t have to rehash all of the results in the Discussion. Instead, plan a small number of subsections (between two and five) within the Discussion, in each of which you state a conclusion, summarize the results that support it, and relate it to previous work in the field. By citing key papers, you acknowledge your debt to your predecessors and avoid being accused of claiming more novelty than is justifiable. This is not the place, though, for a literature review. For example, if you have implicated a gene in a process, you need to be clear on whether this has been done before – but you don’t have to talk about unrelated roles of the gene or its mechanisms of action in other contexts.

These sections can also serve other purposes. You should consider uncertainties and note critical questions that remain unanswered. Acknowledging weaknesses in your argument is not only honest but can be helpful: it is harder for a reviewer to be harshly critical if you have already been self-critical. You can also point out the broader implications of your work and speculate on what the future might hold. Be sparing, though, in claiming that experiments to test your speculations are in progress, as the reviewer or editor might be temped to ask you to resubmit once you’ve done these experiments. And as elsewhere, keep it concise and make sure it furthers the plot.

## The end is near

At this point you have a full draft of the main sections. Once you are fairly satisfied, you’re ready to turn it into a complete manuscript by polishing the figures, writing Figure Legends, Methods and Abstract, and completing the reference list.

Finally, it is time to get feedback from your colleagues. In my lab, we have a practice called 'paper bashing' in which we devote a long lab meeting to going over a paper line by line. Here’s the main lesson I have learned from this painful but invaluable process: almost every time a lab member or other reader points out a problem with a word, sentence, section or conclusion, they are right, and something needs to be done. On the other hand, the particular improvements they suggest are often not the best ones. You have thought about the work more deeply than they have, and are more familiar with the results and the literature, so you are probably better than they are at coming up with useful solutions. In short, use the criticism to highlight points that need attention, but don’t be afraid to use your own judgment in deciding what to do.

It is, after all, your story to tell.

